# From apremilast to JAK inhibitors—salvage treatment strategies for refractory palmoplantar pustulosis: case series

**DOI:** 10.3389/fmed.2026.1743408

**Published:** 2026-02-25

**Authors:** Yufeng Pan, Di Jin, Fanzhang Meng, Hanlu Zhang, Jianong Tang, Cui Guo, Chen Li, Jingjing Ma

**Affiliations:** 1 School of Clinical Medicine, Shandong Second Medical University, Weifang, Shandong, China; 2 Department of Rheumatology, Weifang People’s Hospital, Weifang, Shandong, China; 3 School of Clinical Medicine, Beijing University of Chinese Medicine, Beijing, China; 4 Beijing University of Chinese Medicine, Beijing, China; 5 Department of Dermatology, Tianjin Institute of Integrative Dermatology, Tianjin Academy of Traditional Chinese Medicine Affiliated Hospital, Tianjin, China; 6 Weifang People’s Hospital, Shandong Second Medical University, Weifang, Shandong, China

**Keywords:** apremilast, JAK inhibitors, palmoplantar pustulosis, PDE4 inhibitors, tofacitinib

## Abstract

**Background:**

Palmoplantar pustulosis (PPP) is a chronic inflammatory skin condition characterized primarily by recurrent episodes of blisters and sterile pustules on the palms and soles. It is frequently accompanied by disruption of the skin barrier and intense itching or pain. Currently, there is a lack of standardized treatment protocols for PPP therapy. Traditional first-line therapies primarily include topical corticosteroids, immunosuppressants, and localized phototherapy, which offer limited efficacy and are prone to recurrence. Although apremilast (APR) has been reported for use in refractory PPP, its efficacy varies among individuals. This study aims to explore the value of a rescue therapy strategy switching to JAK inhibitors after APR treatment failure.

**Methods:**

This study is a single-center retrospective case series analysis, enrolling a total of 9 patients with refractory PPP who remained unresponsive to conventional therapy and APR (30 mg twice daily). All patients discontinued APR and initiated JAK inhibitor therapy (tofacitinib 5 mg twice daily). Concurrently record patients’ baseline characteristics, including comorbidities, smoking history, prior medications, baseline skin lesion severity (PPPASI score), skin lesion status before tofacitinib use, and adverse reactions during follow-up.

**Results:**

Our nine patients responded exceptionally well to tofacitinib. By the end of 12 weeks of treatment, the PPPASI scores of all nine patients had significantly decreased. Among them, eight patients achieved PPPASI50 (88.9%), and one patient achieved PPPASI75 (11.1%). The smallest reduction in PPPASI score from baseline was 2.4 points, and the largest reduction was 16.4 points. No serious adverse events were reported during treatment and follow-up.

**Conclusion:**

For refractory PPP patients who fail APR therapy, switching to JAK inhibitors serves as an effective rescue treatment strategy, with most patients achieving remission within a short period and demonstrating good tolerability. This approach offers a viable treatment option for PPP that has proven resistant to conventional therapies and phosphodiesterase-4 (PDE4) inhibitors. Still, its long-term efficacy and safety require validation through large-scale prospective studies.

## 1 Introduction

Palmoplantar pustulosis (PPP) is a chronic inflammatory skin disease characterized primarily by recurrent episodes of blisters and sterile pustules on the palms and the soles. Its course is chronic and recurrent, often accompanied by skin barrier disruption and intense itching or pain, significantly impairing patients’ quality of life (1). Currently, there is no standardized treatment protocol for PPP. Conventional therapies include topical corticosteroids, immunosuppressants, and localized phototherapy, which offer limited efficacy and are prone to recurrence. With advances in research, targeting the immune system has gradually become the core of treatment. Biological agents (e.g., interleukin-23 [IL-23]/interleukin-17 [IL-17] monoclonal antibodies and IL-36 receptor antagonists) and small-molecule drugs (Janus kinase [JAK] inhibitors and phosphodiesterase-4 [PDE4] inhibitors) have gradually been adopted in clinical practice and have achieved satisfactory therapeutic outcomes. In recent years, apremilast (APR) has shown a good response in the treatment of PPP in phase II and III clinical trials for patients with poor response to traditional treatments (2, 3). However, this article reports nine special cases where the efficacy of APR was poor, and after switching to tofacitinib (TOF), significant improvement in palmoplantar lesions was observed.

## 2 Case description

To date, there have been nine cases of PPP that successfully responded to JAK inhibitor therapy after APR treatment failure. Table 1 summarizes the demographic and clinical information for these nine patients. The patient cohort comprised 6 females (66.7%) and 3 males (33.3%), with a mean age of 41.88 years. Six of nine patients (66.7%) were smokers. Nine patients received at least two different systemic treatments before APR, including topical glucocorticoids (GCs), methotrexate (MTX), minocycline (MC), acitretin (Acit), cyclosporine A (CsA), and psoralen-ultraviolet A (PUVA), but had not yielded significant therapeutic efficacy. All patients showed no significant improvement in palmar erythema, sterile pustules, itching, or pain following treatment with apremilast, and some even developed new skin lesions. Among these, 5 cases (55.6%) developed nail lesions, 2 cases (22.2%) exhibited skin fissures and desquamation, and 1 case (11.1%) presented with sterile papules.

**Table 1:** Clinical characteristics and treatment of nine patients with PPP.

Pt	Sex	Age (yrs)	Smoking history	Previous medications before ARP treatment	ARP treatment period (weeks)	Pre-treatment skin lesion status with TOF	TOF time to remission (weeks)	Adverse events
1	M	39	Yes	GCs, PUVA, and TMCs	4	Pustules and scales	12	No
2	M	34	Yes	GCs, CsA, and PUVA	4	Erythema, nail damage, and hyperkeratotic desquamation	16	No
3	M	52	Yes	MTX, Alit, and PUVA	3	Pustular rash, sterile pimples, eruption, and confluent patches.	16	Transient elevation of transaminase levels
4	F	28	Yes	GCs, MC, and TCMs	6	Pustules, skin fissures, and desquamation	12	No
5	F	45	No	GCs and PUVA	4	Nail damage.	20	No
6	F	38	No	GCs and PUVA	3	Nail damage.	12	No
7	F	33	Yes	GCs, Acit, PUVA, and TMCs	5	Pustule, skin fissures, and desquamation.	16	Transient elevation of transaminase levels
8	F	50	No	Alit and PUVA	4	Nail damage.	20	No
9	F	58	Yes	GCs, CsA, Acit, PUVA, and TMCs	3	Nail damage, multiple layers of scales, oyster shell fusion, and diffuse erythematous patches.	12	Diarrhea

Pt, patient; F, female; M, male; PPP, palmoplantar pustulosis; GCs, glucocorticoids; MTX, methotrexate; CsA, cyclosporine A; Acit, acitretin; Alit, alitretinoin; MC, minocycline; PUVA, psoralen-UVA; TCMs, traditional Chinese medicines.

**Figure 1:**
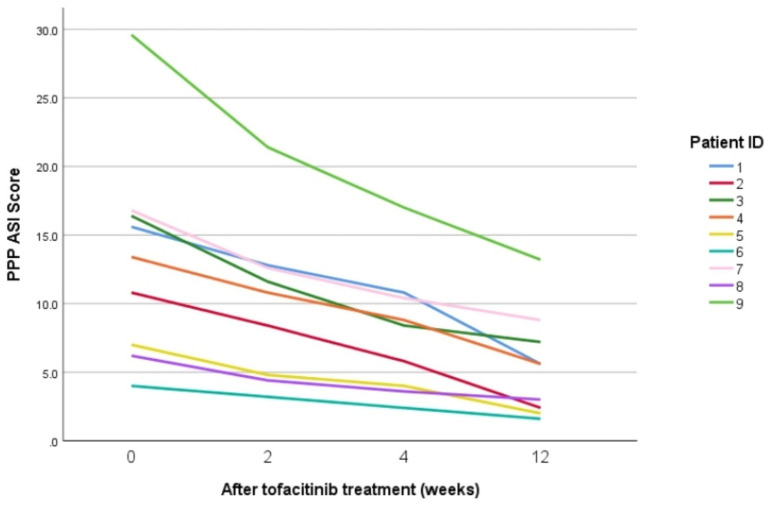
Nine PPPASI scores in 9 PPP patients after 0, 2, 4, and 12 weeks of tofacitinib treatment.

**Figure 2:**
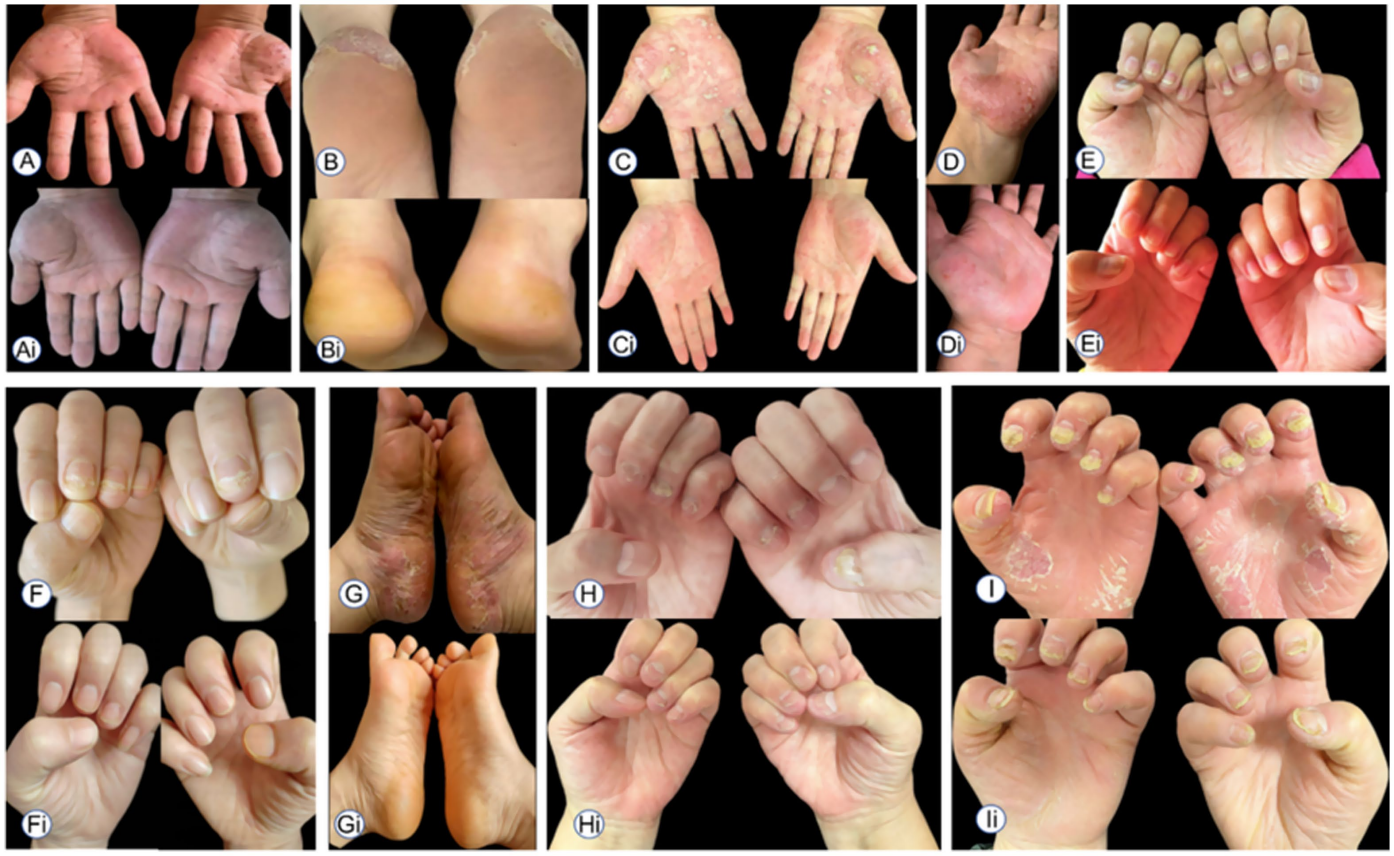
Palmar–plantar skin lesions in PPP patients before tofacitinib treatment **(A–I)**; improvement in palmar–plantar skin condition in PPP patients after 12 weeks of tofacitinib treatment **(Ai–Ii)**.

Following treatment failure with APR, all patients received tofacitinib as a rescue therapy. After 12 weeks of treatment, pustules on the hands and the feet had largely resolved. Nail lesions improved significantly in some patients, with only mild scaling, fissuring, and crusting remaining. By the end of 12 weeks of treatment, the palmoplantar pustulosis psoriasis area and severity index (PPPASI) scores of all nine patients had significantly decreased. Among them, eight patients achieved PPPASI50 (88.9%), and one patient achieved PPPASI75 (11.1%). The smallest reduction in PPPASI score from baseline was 2.4 points, and the largest reduction was 16.4 points. Figure 1 shows the PPPASI scores of the nine patients at 0, 2, 4, and 12 weeks of tofacitinib treatment. Figure 2 shows skin lesions in nine patients with palmoplantar pustulosis who failed APR therapy and subsequently received TOF treatment.

During tofacitinib treatment and follow-up, none of the patients experienced adverse events such as thrombosis, severe infections, or malignancies. One patient developed diarrhea, which was relieved after treatment with montmorillonite powder. Two patients experienced transient increases in transaminases, but the increase did not exceed twice the upper normal limit. They did not receive medication, and the transaminase levels gradually returned to normal. During follow-up, no further abnormalities were observed in liver enzymes.

## 3 Discussion

Currently, the pathogenesis of PPP remains complex and not fully understood. The IL-36 inflammatory pathway, T helper-17 (Th17)/regulatory T (Treg) cell imbalance, local skin immune microenvironment, and neutrophil chemotaxis are considered key drivers of inflammatory progression and the chronic, recurrent nature of PPP (4). IL-36 and the IL-23/Th17 axis synergistically drive inflammation by initiating pro-inflammatory cytokine gene transcription and amplifying the inflammatory cycle via the nuclear factor-κB (NF-κB) signaling cascade. This induces abnormal proliferation of inflammatory keratinocytes and dendritic cells (DCs), which secrete neutrophil chemotactic mediators (IL-36γ, IL-8, and CXCL1) to promote their directional recruitment. This pathway profoundly contributes to and persistently maintains the PPP inflammatory response and high oxidative stress levels, leading to recurrent pustule formation (5–7). Recent research by Daniel McCluskey et al., using single-cell RNA sequencing (scRNA-Seq), revealed that the regulatory Th17 (regTh17) cell subset in patients with PPP exhibits dual Th17 and Treg characteristics. This subset forms an immune-inflammatory network in PPP by enhancing the expression of IL-17F, IL-26, and FOXP3, thereby exacerbating the chronicity and persistence of the inflammatory response (8). The team led by Tran H. Do revealed the intricate activation patterns of T cells and characterized the plasticity of Th17 cells in their transition to a Th2 phenotype. Their work further indicated that smoking may influence the severity of PPP by altering the number and function of Th17/Th1 cells and promoting the phenotypic plasticity of Th17 cells (expression of KLRB1/CD161 and GATA3), leading to disease recurrence (9, 10).

APR is an orally administered small-molecule drug that acts by inhibiting phosphodiesterase-4 (PDE4) activity within the cells. This increases intracellular cyclic adenosine monophosphate (cAMP) levels and activates protein kinase A (PKA), thereby suppressing nuclear factor-κB (NF-κB) activity and downregulating the transcription of pro-inflammatory cytokines and chemokines. This inhibits the abnormal proliferation of keratinocytes, the infiltration of immune cells, and the induction of inflammatory responses in skin and joint tissues (11). The efficacy of APR therapy reaches a plateau over time. A Phase III clinical trial of APR treatment for patients with PPP exhibited significant improvement in clinical symptoms after 16 weeks of treatment. The primary endpoint showed a reduction in the PPPASI total score of 64.3% in the apremilast treatment group. Following an extension to 32 weeks, the PPPASI total score reduction remained at 68.3%, with no further significant improvement observed (2). The “ceiling effect” observed with APR therapy reflects the inherent limitation of single-pathway targeting in fully modulating the complex immune network involving multiple factors. These include immune imbalance, genetic susceptibility, environmental triggers, and disruption of the local skin immune microenvironment, which can result in drug resistance.

Janus kinases (JAKs) are intracellular enzymes that activate signal transducers and activators of transcription (STAT) proteins. Upon binding to membrane receptors, cytokines such as IL-17, IL-23, or IFN-*γ* trigger a JAK phosphorylation cascade. Phosphorylated STAT molecules are subsequently transported to the nucleus via nuclear localization signals, where they participate in the expression of multiple inflammatory cytokine genes. This cascade regulates key cellular processes, including immune and inflammatory responses as well as cell proliferation, thereby playing a key role in the pathogenesis of immune-mediated diseases (12, 13). Tofacitinib, as a pan-JAK inhibitor, directly targets Janus kinases, blocks IL-36 pathway-induced STAT3 phosphorylation, suppresses Th17 and Th2 cell differentiation, restores the expression of anti-inflammatory cytokine genes, and reduces the release of inflammatory cytokines such as IL-7, IL-17A, and IL-22, thereby decreasing immune cell activation and infiltration (14–16). Simultaneously, it can also inhibit the transition of Th17 cells to Th2 cells by reducing the secretion of pro-inflammatory cytokines such as IL-4 and IL-6. Several case reports and series have confirmed that tofacitinib is effective in patients with refractory PPP. A retrospective analysis included 6 cases of PPP with poor responses to conventional therapy. All patients achieved a ≥ 50% reduction in PPPASI scores after 4 weeks of treatment, with half of the cases (3/6) achieving a ≥ 80% reduction. At 12 weeks, 83.3% (5/6) of patients maintained a ≥ 80% reduction in PPPASI scores, and no serious adverse events were reported (17). Tofacitinib effectively suppresses the progression of inflammation in autoimmune diseases across multiple levels and dimensions. These mechanisms explain the primary reason for tofacitinib’s efficacy in PPP and support its potential as a therapeutic agent for refractory disease.

The majority of patients experience suboptimal efficacy with conventional treatments or biologics such as adalimumab and ustekinumab, with some even exhibiting paradoxical worsening of PPP (18). Nearly all patients who subsequently switched to the pan-JAK inhibitor tofacitinib experienced significant improvement in their skin lesions, with no serious adverse events observed (17). Regarding therapeutic options, selective JAK inhibitors (upadacitinib and baricitinib) are being increasingly used in the treatment of PPP by inhibiting pro-inflammatory cytokine signaling, thereby modulating innate and adaptive immunity (19). Neda et al. reported five patients with PPP treated with upadacitinib. At the 12-week treatment endpoint, three patients achieved PPPASI50, two achieved PPPASI75, and one achieved PPPASI90, with no serious adverse events occurring (20).

The most common adverse reactions associated with JAK inhibitors primarily involve an increased risk of infections, such as upper respiratory tract infections and urinary tract infections. Reports also indicate increased risks of skin reactions, hair loss, dyslipidemia, anemia, leukopenia, and cardiovascular events, which are dose-dependent (21). During treatment with tofacitinib 5 mg twice daily in nine patients with PPP in this study, no serious adverse reactions were observed, and the drug was well-tolerated. These patients remain under follow-up.

## 4 Conclusion

PPP involves multiple complex and dynamically changing pathological mechanisms. This case report suggests that PDE4 inhibitor therapy alone fails to yield significant efficacy, and pathological alterations in PPP may not be fully resolved through monotherapy. JAK inhibitors may represent an ideal therapeutic option for targeting the PPP multi-signaling pathway and may also serve as a rescue strategy following failure of PDE4 inhibitor therapy. However, further large-scale randomized controlled trials are still needed to evaluate the efficacy and safety of JAK inhibitors in PPP treatment and to determine the optimal administration regimens. Further clarification of the synergistic interactions among multiple key signaling pathways involved in the pathogenesis of PPP is required to identify new effective therapeutic strategies for PPP.

## Data Availability

The original contributions presented in the study are included in the article/supplementary material, further inquiries can be directed to the corresponding authors.
